# Fundamental and Applied Aspects of Physics in Low-Dimensional Systems

**DOI:** 10.3390/nano16040242

**Published:** 2026-02-13

**Authors:** Orion Ciftja

**Affiliations:** Department of Physics, Prairie View A&M University, Prairie View, TX 77446, USA; ogciftja@pvamu.edu

It is my pleasure as Guest Editor to introduce this Special Issue reprint, which brings together eleven high-quality contributions exploring both fundamental physics and practical applications in low-dimensional systems. Low-dimensional materials and structures display a remarkable range of unique behaviors. The distinctive characteristics of these systems lead to phenomena that are often absent in bulk materials. The papers in this collection reflect diverse theoretical, computational, and experimental advances, ranging from analytical modeling and numerical simulations to sophisticated experimental techniques, all of which deepen our understanding of these phenomena. Together, they highlight how subtle changes in geometry, dimensionality, or composition can drastically influence material properties and provide pathways to control and exploit these effects for technological applications. By covering topics that span fundamental studies of electron interactions, molecular dynamics, and quantum mechanical modeling, as well as applied research and materials functionalization, this Special Issue provides a comprehensive snapshot of current research in low-dimensional physics. The contributions collectively illustrate the interplay between theory and experiment, revealing both the richness of the underlying physics and the potential for translating these insights into innovative devices and functional materials.

The studies included in this Special Issue are particularly important because low-dimensional systems form the foundation for a wide range of emerging technologies and allow the exploration of novel physical phenomena that are absent in bulk materials. In these systems, quantum confinement, surface and interface effects, and reduced symmetry can profoundly alter electronic, optical, magnetic, thermal, and mechanical properties. Such modifications give rise to unique behaviors, including enhanced electron mobility, tunable band gaps, strong excitonic interactions, spin-dependent effects, and exceptional mechanical strength, which are critical for understanding fundamental physics and for designing advanced functional materials. Insights into these phenomena are essential not only for condensed matter physics but also for the development of next-generation devices in nanoelectronics, optoelectronics, spintronics, photonics, sensing, and energy harvesting. Low-dimensional materials, such as nanowires, quantum dots, nanoribbons, graphene derivatives, and carbon nanotubes, provide versatile platforms to manipulate charge, spin, and heat at the nanoscale. By combining theoretical modeling, computational simulations, and experimental investigations, the contributions in this Special Issue illuminate the interplay between geometry, dimensionality, and interactions, showing how subtle structural modifications can significantly influence material behavior. Collectively, these studies highlight the richness and versatility of low-dimensional systems, demonstrating how a deep understanding of their properties can guide the design of novel materials and devices for a broad range of applications, from high-performance electronics and sensors to energy conversion and catalysis.

The first article by Ciftja [1], develops an exact analytical expression for the electrostatic interaction potential between a uniformly charged square nanoplate and a coplanar nanowire, a model that is useful for classical descriptions of finite two dimensional (2D) systems and hybrid charged nanosystems. The second article by Wang et al. [2] experimentally investigates the physical mechanism of selective healing of nanopores under laser irradiation and plasma. This approach establishes a model for enhanced surface hardness and resistance to cracking in condensed matter systems by controlling nanoscale features. Following this, the third article by Vrinceanu [3] deals with the quantum states of a 2D dipole. The work develops improved numerical methods that account for the correct asymptotic behavior of wave functions, yielding accurate ground and excited states of 2D dipole systems relevant to nanoelectronics. The fourth paper by Mikhailov [4] is a contribution on the fractional quantum Hall effect and presents an exact diagonalization framework study, challenging conventional descriptions of the Laughlin state and offering insights into the nature of ground and excited states in interacting 2D electron systems under strong magnetic fields. The fifth paper by Qiao et al. [5] is an investigation into nanocrystalline composite deformation at cryogenic temperatures. The work combines experimental measurements and theoretical modeling to understand fracture and plasticity in layered nanomaterials, relevant to low dimensional alloy systems. The sixth work on graphitic carbon nitride catalysis by Okolie et al. [6] reports its capabilities of catalyzing the reduction of the azo bond by hydrazine to two amines under visible light, highlighting the potential use of layered 2D semiconductors for environmental and chemical applications. The seventh paper by Gao et al. [7] examines the stability of ultraviolet-defluorination driven crosslinked carbon nanotubes, revealing how strain and lattice curvature influence thermal stability and potential applications of carbon nanotube networks. The eighth paper by Liu et al. [8] is a quantum mechanical second-order Møller–Plesset perturbation theory study of the electronic effect of nonplanarity on fullerene $C_{60}$. Advanced electronic structure calculations reveal how nonplanarity and hybridization influence the electronic properties of fullerene, with implications for ${\mathrm{CO}}_{2}$ reduction catalysts. The ninth paper by Ciftja et al. [9] introduces a model and calculates the energy bounds for a 2D system of electrons localized in concentric rings. It uses semi-classical and numerical methods to investigate equilibrium energy configurations, offering insights into interacting electron systems with circular symmetry. The tenth paper by Liu et al. [10] shifts attention to molecular magnetism. This work represents a study of coordinate quasi-double bonds in Co(II) complexes and provides theoretical insights into bonding and magnetic properties relevant for single-molecule magnet design. The last paper by Tesema [11] explores oscillatory photoluminescence in Langmuir–Blodgett quantum dot films, uncovering strain-assisted atomic transfer effects that drive dynamic optical responses in close-packed assemblies

Together, these papers span a broad range of subjects, from quantum dot optics and molecular magnetism to quantum Hall theory, nanomaterial stability, and nanoscale mechanical behavior. This reprint serves as a snapshot of cutting edge research in low-dimensional physics and is intended as a valuable resource for researchers, educators, and students engaging with both foundational questions and emerging applications. I extend my gratitude to all contributing authors for their excellent work and to the reviewers and editorial team for their support in bringing this Special Issue to fruition.

## References

[1] Ciftja O. (2023). Interaction potential between a uniformly charged square nanoplate and coplanar nanowire. Nanomaterials.

[2] Wang Z., Ushakov I.V., Safronov I.S., Zuo J. (2024). Physical mechanism of selective healing of nanopores in condensed matter under the influence of laser irradiation and plasma. Nanomaterials.

[3] Vrinceanu D. (2024). Accurate quantum states for a two-dimensional dipole. Nanomaterials.

[4] Mikhailov S.A. (2024). Toward a new theory of the fractional quantum Hall effect. Nanomaterials.

[5] Qiao J., Ushakov I.V., Safronov I.S., Oshorov A.D., Wang Z., Andrukhova O.V., Rychkova O.V. (2024). Physical mechanism of nanocrystalline composite deformation responsible for fracture plastic nature at cryogenic temperatures. Nanomaterials.

[6] Okolie M.C., Ollordaa G.G., Ramidi G.R., Yan X., Quan Y., Wang Q., Li Y. (2024). Graphitic carbon nitride catalyzes the reduction of the azo bond by hydrazine under visible light. Nanomaterials.

[7] Gao Y., Islam M.T., Otuokere P.U., Pulikkathara M., Liu Y. (2024). The stability of UV-defluorination-driven crosslinked carbon nanotubes: A Raman study. Nanomaterials.

[8] Liu Y., Gao Y., Altalhi T., Liu D.-J., Yakobson B.I. (2024). A quantum mechanical MP2 study of the electronic effect of nonplanarity on the carbon pyramidalization of fullerene C_60_. Nanomaterials.

[9] Ciftja O., Batle J., Abdel-Aty M., Hafez M.A., Alkhazaleh S. (2024). Model and energy bounds for a two-dimensional system of electrons localized in concentric rings. Nanomaterials.

[10] Liu Y., Massoud S.S., Starovoytov O.N., Altalhi T., Gao Y., Yakobson B.I. (2025). Quantum mechanics MP2 and CASSCF study of coordinate quasi-double bonds in cobalt(II) complexes as single molecule magnets. Nanomaterials.

[11] Tesema T.E. (2025). Light-driven quantum dot dialogues: Oscillatory photoluminescence in Langmuir–Blodgett films. Nanomaterials.

